# Evaluation of Rational Drug Use for Acute Pharyngitis Associated with the Incidence and Prevalence of the Disease at Two Community Health Centers in Indonesia

**DOI:** 10.3390/scipharm85020022

**Published:** 2017-04-28

**Authors:** Cindra T. Yuniar, Kusnandar Anggadiredja, Alfi N. Islamiyah

**Affiliations:** 1 Pharmacology-Clinical Pharmacy Research Group, School of Pharmacy, Institut Teknologi Bandung, Bandung 40132, Indonesia; kusnandar@fa.itb.ac.id; 2 School of Pharmacy, Institut Teknologi Bandung, Bandung 40132, Indonesia; alfi@s.itb.ac.id

**Keywords:** acute pharyngitis, rational use of drugs, incidence, prevalence

## Abstract

According to Indonesia’s Result of Basic Health Research of 2013, prevalence of acute respiratory infection in 2007 and 2013 were not significantly different (25.5% and 25.0%, respectively). Identifying the cause of acute pharyngitis is a key point in determining the optimal treatment. The main purpose is to evaluate the rational use of drugs and its irrational impact as well as the correlation of the drug use with the incidence and prevalence of acute pharyngitis. This study was a descriptive and observational study, carried out retrospectively and concurrently at two community health centers located in Bandung and Cimahi, Indonesia. There was overprescription of antibiotics in 80.01% of prescription cases, with a total of 8.98% being non-treatment option, and 62.43% being irrational use of corticosteroids. The incidence and prevalence of acute pharyngitis at one health center in Bandung were 2.45% and 2.31%, respectively, with an irrationality rate of 83.82%. Those recorded at one health center in Cimahi were 2.11% incidence and 2.00% prevalence with an irrational rate of 91.29%. It can be concluded that there is still an irrational use of medicines in the treatment of acute pharyngitis in community health centers. The higher incidence and prevalence might indicate the declining quality of health services.

## 1. Introduction

Acute respiratory infections (ARI) are one of the most common diseases, accounting for one of the main causes of patient visits to community health centers (40–60%) and hospitals (15–30%). There are 156 million new episodes per year globally, of which 151 million (96.7%) occur in developing countries. Indonesia is one of the top five countries with the highest ARI cases with as many as 6 million episodes per year [1]. Results of the National Basic Health Research of the Indonesian Ministry of Health showed that the prevalence of ARI in 2013 (25.0%) has not changed since 2007 (25.5%), indicating that the control and management of ARI is still not optimal [2]. Furthermore, acute pharyngitis, which is part of ARI, accounts for an estimated 2–5% of patient visits to health facilities [3,4,5]. In 2004, acute pharyngitis was one of the top ten diseases in outpatient visits in Indonesia, (1.5% or 214,781 patients). About 40 million people visited health facilities each year [6,7].

Acute pharyngitis is an acute infection or inflammation in the mucosa of the pharynx, and generally extends into the surrounding tissue. It is commonly caused by viruses (40–60%) and bacteria (5–40%). *Streptococcus pyogenes* is the most common pathogen found in patients suffering acute pharyngitis due to bacteria [3,7]. Thus, antibiotic therapy is generally not recommended, and this disease can heal even without intervention. Identifying the cause of acute pharyngitis is a key point in determining the optimal treatment for the patient.

The World Health Organization (WHO) states that rational use of medicines requires that “patients receive medications appropriate to their clinical needs, in doses that meet their own individual requirements, for an adequate period of time, and at the lowest cost to them and their community” [8]. Also, the Ministry of Health of Indonesia states that use of a drug is said to be rational if it meets the “appropriate diagnosis, indications, drug selection, dosage, route, interval, and duration of administration, wary of side effects, precise assessment of the conditions, right of information, proper follow-up and delivery of drugs, allowing to comply to the regimens, as well as the guaranteed of it safety, efficacy, quality and available at any time at an affordable price” [9]. The WHO has estimated that more than 50% of drugs were prescribed, given, and sold in a way that was inappropriate, ineffective, and inefficient, and also estimated that 50% of drug used inappropriately [10]. Inappropriate use of antibiotics lead to resistance and has a greater impact on the reduction of the efficacy of antibacterials in the human population.

Incidence is defined as the number of new cases in a particular population, while prevalence is the number of both old and new cases in a population. Incidence measures the rate of occurrence of new cases of a disease or condition, calculated as the number of new cases of a disease or condition in a specified time period divided by the size of the population under consideration who are initially disease**-**free. Prevalence is a frequently used measure of how commonly a disease or condition occurs in a population; it measures how much of some disease or condition there is in a population at a particular point in time. Estimating the incidence and prevalence of a disease is crucial for evaluating the current and projected future unmet medical need for drugs. Epidemiologic studies are useful for identifying patterns of health care utilization. They can provide estimates of the magnitude of risk related to a particular level of dose and so can be used in the evaluation of appropriate microbiological quality guideline levels or standards. Epidemiological methods can quantify the probability that observed relationships occurred by chance factors and they also have the potential to control for other risk factors and/or confounders of the outcome illness being studied [11,12]. This study aimed to evaluate the rational use of drugs and its irrational impact as well as the correlation of the drug use with the incidence and prevalence of acute pharyngitis.

## 2. Materials and Methods

This study is a descriptive and observational study involving measurements of the incidence and prevalence rates of acute pharyngitis and the evaluation of the rational use of drugs. It was carried out retrospectively and concurrently from October 2014 to May 2015 at two community health centers in Bandung and Cimahi, Indonesia. The authors observed the participants without providing any interventions related to the treatment prescribed and then the results are presented descriptively. The participants were patients with acute pharyngitis aged ≥3 years. Patients who were lost to follow-up or have comorbidity with other infectious diseases requiring antibiotic therapy were excluded. Patients were grouped based on the etiology of acute pharyngitis, which is determined by the Centor score.

Data were obtained from medical records, interviews, and the results of follow-up visits. Data from medical records include patient’s identity, diagnosis, history and treatment of acute pharyngitis. The interviews were conducted to determine the initial condition of the patient. Follow-up visits were performed seven days later to evaluate the outcome of therapy, compliance, and impact of the treatment. Population data, as well as reference incidence and prevalence rates of acute pharyngitis, were obtained through reports from the Ministry of Health of Indonesia and related institutions.

The rationality of drug use is defined by prescribing indicators that consist of appropriate indication, drug selection, dosage, route, intervals, duration of administration, assessment of the patient’s condition, and patient compliance. The outcome of therapy was assessed by the patient’s condition in the last visit compared to their initial condition. Data were analyzed descriptively to conclude the event of irrational use of drugs, the proportion of antibiotic prescriptions, the impact of irrational use of drugs, and correlation of drug use with the incidence and prevalence of acute pharyngitis.

## 3. Results

### 3.1 Causes of Acute Pharyngitis Based on the Centor Criteria

The total number of patients diagnosed with acute pharyngitis in a community health center in Bandung and Cimahi were 1083 and 995 patients, respectively. As many as 1641 patients met the inclusion criteria; 733 patients (44.67%) were aged 15–44 years, 504 patients (30.71%) were aged 3–14 years, and 404 patients (24.62%) were >44 years old. The patients were grouped based on the etiology of acute pharyngitis. In this study, a modified Centor score, which also considers the patient’s age, was calculated for all patients who met the inclusion criteria (Table 1).

Patients with a score of zero or 1 was at very low risk (<10%) of Streptococcal pharyngitis, and those with a score of 4 or higher were at high risk (>50%). Patients with a score of 2–3 should be tested using rapid antigen detection test (RADT) or throat culture, with positive results warranting antibiotic therapy. However, in clinical practice in almost all health centers in Indonesia, these tests are not performed due to limited facilities. Thus, patients with a Centor score of 2–3 were grouped separately. Based on the results above, the incidences of the causes of acute pharyngitis in both health centers have similarities (Table 2). As many as 226 patients (13.77%) were diagnosed with streptococcal pharyngitis, 1179 patients (71.85%) were infected by viruses, and the causes of acute pharyngitis in 236 patients (14.38%) could not be determined. Also, respiratory infection contributed 32% of visits at both health centers, of which 9.58% of them were patients with acute pharyngitis. More than 50% of the antibiotics used in the health centers were given to patients with respiratory infection, and 28.96% were given to acute pharyngitis patients. 

### 3.2 Drug Use Evaluation

The therapy goal of acute pharyngitis is to improve the signs and symptoms, minimize side effects, and prevent transmission and complications. Antibiotics were only indicated in patients with bacterial pharyingitis. We found that there were indications of irrational use of antibiotics in the treatment of acute pharyngitis (Table 3). Inappropriate indication (87.43%), inappropriate drug selection (0.09%), and inappropriate duration of administration were found in almost all patients who received antibiotics. Additionally, no antibiotics were prescribed to 2.97% patients suspected of being infected by bacteria.

**Figure 1:**
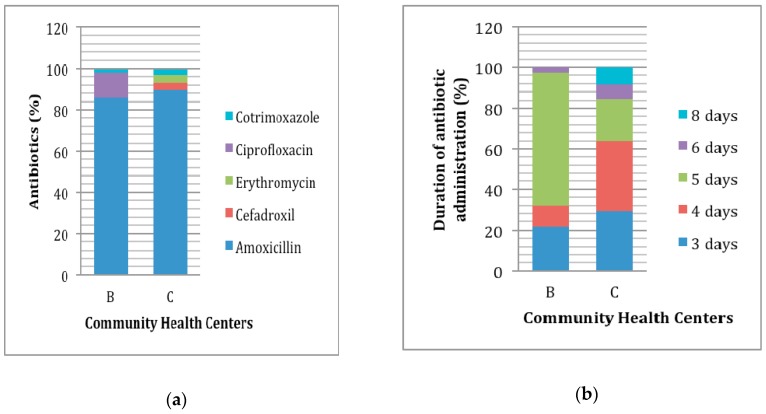
Use of antibiotic in the treatment of acute pharyngitis patients: (**a**) Antibiotic that was prescribed; (**b**) Duration of antibiotic administration; B: health center in Bandung; C: health center in Cimahi.

As much as 88.24% acute pharyngitis patients received antibiotics, but only 8.23% of the patients showed indications warranting their use. Thus, it can be concluded that there was an 80.01% overprescribing of antibiotics in the treatment of acute pharyngitis in the two health centers. Amoxicillin, one of the first-line antibiotics used for acute pharyngitis caused by bacteria, was the most common antibiotic prescribed (Figure 1a). A total of 8.98% antibiotics was non-treatment option (ciprofloxacin: 6.28%, cotrimoxazole: 2.26%, and thiamphenicol: 0.44%). The duration of antibiotic administration for acute pharyngitis is 6–10 days for adults and 10 days for children. In this study, the appropriate duration of treatment was only found in 0.57% of patients (Figure 1b); most of the patients (50%) received antibiotics for 5 days. 

In this study, it was found that corticosteroids were prescribed to about 20–30% of patients. Out of these prescriptions, irrational use of corticosteroids was found, including inappropriate dosage, inappropriate interval of administration, and inappropriate duration of administration. Also, a majority of pediatric patients received overdose of corticosteroids. The Ibrahim Adjie health center in Bandung tends to prescribe dexamethasone with a prolonged interval (12 h), while the South Cimahi health center tends to prescribe dexamethasone with a shorter duration of administration (2 days).

Besides examining medical records, interviews were conducted concurrently with 108 acute pharyngitis patients, and follow-up visits were performed seven days later to examine the patients’ final condition (Table 4). The results show that as many as 31 patients (28.70%) were lost to follow-up. A total of 41 patients (56.94%) who received antibiotics showed improved health, while 28 patients (38.89%) still had complaints, and 3 patients’ (4.17%) conditions worsened. In the meantime, 2 patients (40%) who did not receive antibiotics showed improved health, while another 2 patients (40%) still had complaints, and 1 patient’s (20%) health worsened. Overall, 38.96% (30 patients) recovered in ≤3 days, 14.29% (11 patients) recovered within 4–6 days, and 2.60% (2 patients) recovered in more than 6 days. Furthermore, the result of the follow-up also showed that 58.06% patients adhered to the regimen of antibiotics prescribed by doctors and 41.94% of patients did not comply. Reasons for non-compliance included forgetting to take the antibiotics and stopping altogether. Patients who stopped taking the antibiotics stated side effects (6.54%: dizziness, headache, weakness, stiffness), discomfort in the drug form, or their ignorance as reasons for stopping. Adverse effects of therapy were reported in 5 patients who receive an antibiotic (Table 5). The majority of patients experienced side-effects in the form of headaches (37.5%), and subsequently in the form of dizziness, headaches, weakness, stiffness, shoulder pain, and heartburn.

**Figure 2:**
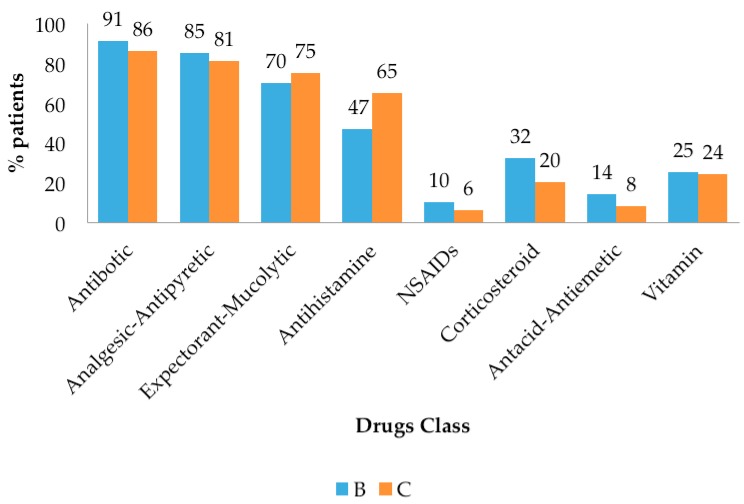
Drugs prescribed to acute pharyngitis patients. NSAIDs = Nonsteroidal anti-inflammatory drugs.

The health centers in Bandung and Cimahi showed the same prescribing pattern. At least 50% of acute pharyngitis patients were treated with a combination of antibiotics, analgesic-antipyretic, expectorant-mucolytic, and antihistamines (Figure 2). Symptomatic drugs prescribed include corticosteroids (dexamethasone: 24.92%, prednisone), analgesic-antipyretics (paracetamol: 82.55%, antalgin), expectorants-mucolytics (glyceryl guaiacolate: 37.19%, ambroxol, black cough syrup), antihistamine (chlorpheniramine maleate: 54.55% loratadine), nonsteroidal anti-inflammatory drugs (NSAIDs) (diclofenac: 3.22%, ibuprofen, mefenamic acid, piroxicam), antacid-antiemetics (antacid: 10.24%, domperidone, metoclopramide), vitamins (B complex: 10.31%, C, B1, B6, B12, multivitamin).

### 3.3 Incidence and Prevalence Calculation

The incidence and prevalence of acute pharyngitis was calculated based on the patient’s visit. The total population in the Bandung and Cimahi health center was 44,608 and 35,833 people, respectively. Meanwhile, there were 1069 new cases of acute pharyngitis with a total of 1032 patients in Bandung, and 979 new cases with a total of 945 patients in Cimahi. Thus, the incidence and prevalence of acute pharyngitis at the Bandung health center were 2.45% and 2.31%, and 2.11% and 2.00% in Cimahi, respectively.

## 4. Discussion

Indonesia’s health ministry stipulated that one of the indicators of the rational use of drugs in the handling of non-pneumonia ARI, including acute pharyngitis, is limiting the number of antibiotics prescribed to 20% of the total number of patients [9]. However, as much as 88.24% acute pharyngitis patients in the health centers in Bandung and Cimahi received antibiotics, with a total of 8.98% of antibiotics prescribed for non-treatment option. Therefore, it can be concluded that the use of antibiotics in the treatment of acute pharyngitis was not rational. Irrational used of antibiotics include inappropriate indication, inappropriate drug selection, and inappropriate duration of administration. Furthermore, it was also found that some patients suspected of being infected by bacteria did not receive any antibiotics.

Signs and symptoms of acute pharyngitis caused by bacteria, viruses, and other microbes generally overlap and difficult to distinguish. Identifying the cause of acute pharyngitis is a key point in determining the optimal treatment. Although the diagnosis of the etiology of acute pharyngitis depends on the results of laboratory tests such as RADT or throat culture, a scoring system based on the clinical manifestations has been developed to predict the risk of infection of *S. pyogenes* in acute pharyngitis patients [13]. The Centor score is a scoring method that can be used to estimate the risk of *S. pyogenes* infection as the cause of acute pharyngitis based on clinical signs and symptoms [14]. This method is essential especially in developing countries such as Indonesia where laboratory tests in clinical practice in almost all health centers is not performed due to the limited facilities. The original Centor score used four signs and symptoms to estimate the probability of streptococcal pharyngitis in adults with a sore throat [15]. The signs and symptoms include the absence of cough (1 point), swollen and tender anterior cervical nodes (1 point), a body temperature of more than 38 °C (1 point), and tonsillar exudates or swelling (1 point). The score was later modified by adding age and validated in adult and children patients (age 3–14 years: 1 point, age 15–44 years: 0 point, age >44 years: −1 point) [16]. This scoring system has been validated in 206,870 patients of age >3 years with a sore throat. The cumulative score specifies the likelihood of streptococcal pharyngitis and the need for antibiotics [17]. 

Antibiotics are only indicated for patients suffering pharyngitis caused by bacteria. Empirical antibiotic therapy may be considered in patients with a Centor score of 4 or higher who are at high risk (>50%) of streptococcal pharyngitis. The selection of antibiotics prescribed requires consideration of the effectiveness, spectrum of activity, safety, dosing schedule, cost, and compliance issues of the antibiotic.

Penicillin, penicillin congeners (ampicillin or amoxicillin), clindamycin, and certain cephalosporins and macrolides are effective against streptococcal pharyngitis. Based on cost, narrow spectrum of activity, safety, and effectiveness, penicillin is recommended by the American Academy of Family Physicians (AAFP) [18], the American Academy of Pediatrics (AAP) [19], the American Heart Association (AHA) [20], the Infectious Diseases Society of America (IDSA) [21], and WHO for the treatment of streptococcal pharyngitis [22]. Penicillin is often substituted by amoxicillin oral suspension due to its better taste. Five out of eight studies have proven that amoxicillin can eradicate >85% *S. pyogenes*, which can be considered equivalent to penicillin, which can eradicate >92% of these microbes [14]. Erythromycin or first-generation oral cephalosporins such as cephalexin are second-line antibiotics that can be used if the patient is allergic to penicillin, or if the symptoms persist after therapy with first-line antibiotics. Quinolone, tetracycline and sulfonamides are not recommended for the treatment of streptococcal pharyngitis. Tetracycline has a high prevalence for development of resistance, and sulfonamides and trimethoprim-cotrimoxazole cannot eradicate the pathogen in streptococcal pharyngitis. Additionally, old generation fluoroquinolones such as ciprofloxacin has limited activity against *S. pyogenes* [23]. It is often indicated as a first line treatment for urinary tract infection [24] but it was as effective as amoxycillin with a successful outcome in 81% and 82% of cases respectively [25]. In this study, ciprofloxacin (12.36%) was prescribed by physicians for upper respiratory tract infection.

Despite the low incidence of acute pharyngitis caused by bacteria, prescription of antibiotics is exceptionally high. A study in America (2005) reported that 53% of children with acute pharyngitis received antibiotics, far exceeding the prevalence of streptococcal pharyngitis. Meanwhile, in Brazil (2014), it was found that 73% of adult patients with acute pharyngitis received antibiotics [24]. In Indonesia, through this study, it can be concluded that respiratory infections contributed to 32% of visits to the health centers. More than 50% of the antibiotics in health centers were used for this treatment, where about 30% of those antibiotics were given to acute pharyngitis patients. About 80% of acute pharyngitis patients received antibiotics along with the inappropriate duration of administration, which would greatly contribute to antibacterial resistance. In the community setting in the Bandung region, ampicillin resistance was observed most frequently (34%), followed by trimethoprim/sulfamethoxazole resistance (29%) and chloramphenicol resistance (15%). Resistance to ciprofloxacin, gentamicin and cefotaxime occurred less than 100 times [26].

Acute pharyngitis is expected to be the main cause of inappropriate use of antibiotics in clinical practice. Antibiotics used in the treatment of respiratory infection, including acute pharyngitis, are thought to be the biggest contributor to the development of resistance.

The standard duration of antibiotic administration for acute pharyngitis is 6–10 days for adults and 10 days for children, but patient compliance in this regard was generally low [7]. This study found that 41.94% of the patients did not comply with the antibiotic regimens prescribed. Studies of antibiotic therapy for acute pharyngitis with a shorter duration (3–6 days) have been performed, but the effectiveness and clinical application of this strategy is still debated [27,28,29]. The administering of antibiotics for acute pharyngitis with the proper duration is rare or almost do not occur in clinical practice in Indonesia. The appropriateness of the duration of administration only occurred in 0.57% of patients, with most patients (50%) being given antibiotics for 5 days. 

Furthermore, the use of corticosteroids in acute pharyngitis patients remained controversial [7,14,31,32]. Sore throats, the main complaint by patients in most cases of acute pharyngitis, usually occur due to inflammation of the pharynx. The administration of anti-inflammatory agents such as corticosteroids to treat the inflammation is generally thought to quickly relieve pain and accelerate clinical improvement. Corticosteroids can be given in the form of dexamethasone with a regiment of 3 × 0.5 mg dose for 3 days in adults and 0.01 mg/kg divided into 3 doses for 3 days in children [7].

Based on these results, it can be predicted that one of the effects of the irrational use of antibiotics was the increased potential of noncompliance that can lead to the emergence of bacterial resistance. Overprescribing antibiotics could increase the risk of side effects in patients, which was seen at 6.45% of patients taking antibiotics. Irrational use of antibiotics by physicians can have an impact on the quality of medicine and health services, increasing the problem of resistance, waste costs, increasing the risk of side effects, as well as the psychosocial impact. In Indonesia, prescription written by a physician or dentist or veterinary in community and hospital settings [33], so that education and health promotion to the physicians about appropriate use of antibacterial is important.

The health centers in Bandung and Cimahi studied in this research did not differ in the incidence and prevalence rates of acute pharyngitis, so that it can be concluded that they have the same quality of health care in the treatment of this disease. The calculations show an increase in incidence and prevalence of acute pharyngitis of 0.5–1% in ten years, which might indicate the declining quality of health services. However, it must be noted that treatment is only one of the factors that can affect the quality of health services, including the values of incidence and prevalence. The incidence and prevalence of a disease can be affected by various other factors, such as mortality rate, number of healing, treatment setting, and the success of disease prevention. 

The rational use of drugs is aimed to facilitate the access of the public to obtain drugs at affordable prices, preventing the impact of inappropriate use of drugs that may be harmful, and improve compliance. Prudence in prescribing antibiotics for acute pharyngitis in health centers is also expected to improve the effectiveness and efficiency of drug expenditures. Each physician has the right to determine the type of drug that is most appropriate for their patients. However, if it is not controlled or not based on acceptable scientific research, then it would risk incurring the irrational use of drugs. Improving the rational use of drugs requires the involvement all elements of healthcare professionals, especially pharmacists as a co-physician in prescribing medication.

## 5. Conclusions

Irrational use of drugs in the treatment of acute pharyngitis was found in two community health centers in Bandung and Cimahi, Indonesia. The irrational use of drugs mostly occurred in the use of antibiotics and corticosteroids. Also, the increase in disease incidence and prevalence might indicate a decline in health services quality.

## Figures and Tables

**Table 1:** The modified Centor score of acute pharyngitis patients in a community health center in Bandung and Cimahi, Indonesia.

Centor	Risk of Streptococcus Infection	Health Center in Bandung	Health Center in Cimahi
		∑ Patients (%)	∑ Patients (%)
≤0	1–2.5%	209 (25.30)	161 (19.68)
1	5–10%	202 (24.46)	268 (32.76)
2	11–17%	437 (52.91)	424 (51.83)
3	28–35%	213 (25.79)	121 (14.79)
≥4	51–53%	19 (2.30)	3 (4.03)
Total patients		826	818

**Table 2:** The causes of acute pharyngitis in patients.

Etiology	One Health Center in Bandung	One Health Center in Cimahi
	∑ Patients (%)	∑ Patients (%)
Bacteria	136 (16.52)	90 (11.00)
Virus	564 (68.53)	615 (75.18)
Cannot be determined	123 (14.95)	113 (13.81)
Total patients	826	818

**Table 3:** Irrational use of drugs in the treatment of acute pharyngitis.

Criteria	Antibiotic Therapy (∑ Patients, %)		Corticosteroid Therapy * ∑ Patients (%)
	Health Center in Bandung	Health Center in Cimahi	
Inappropriate indication	627 (83.82)	639 (91.29)	x
Inappropriate drug selection	10 (1.34)	3 (0.43)	x
Inappropriate dosage	x	x	55 (15.19)
Inappropriate route of administration	x	x	x
Inappropriate intervals of administration	x	x	134 (37.02)
Inappropriate duration of administration	109 (90.08)	54 (88.52)	37 (10.22)
Inappropriate assessment of the patient’s condition	x	x	x

Note: * only in health center in Bandung.

**Table 4:** Outcome of the therapy of acute pharyngitis patients.

Score Centor	Outcome Therapy				
	Improved			Unimproved	
	≤3 days	4–6 days	≥7 days	Persistent	Worsen
Antibiotic	29	10	2	28	3
Without Antibiotic	1	1	0	2	1
Total	30	11	2	30	4

**Table 5:** Adverse effects of the therapy in patients receiving antibiotics.

Adverse Effect	Incidence (%)
Dizziness	1 (12.5)
Headache	3 (37.5)
Weakness	1 (12.5)
Stiffness	1 (12.5)
Shoulder pain	1 (12.5)
Heartburn	1 (12.5)
